# Generation and Characterization of a Transgenic Pig Carrying a DsRed-Monomer Reporter Gene

**DOI:** 10.1371/journal.pone.0106864

**Published:** 2014-09-04

**Authors:** Chih-Jen Chou, Shao-Yu Peng, Mei-Han Wu, Cho-Chen Yang, Yu-Sheng Lin, Winston Teng-Kui Cheng, Shinn-Chih Wu, Yao-Ping Lin

**Affiliations:** 1 Institute of Biotechnology, National Taiwan University, Taipei, Taiwan; 2 Department of Radiology, Taipei Veterans General Hospital, Taipei, Taiwan; 3 Department of Animal Science, National Chiayi University, Chiayi, Taiwan; 4 Department of Animal Science and Technology, National Taiwan University, Taipei, Taiwan; 5 Department of Animal Science and Biotechnology, Tunghai University, Taichung, Taiwan; 6 Division of Nephrology, Department of Medicine, Taipei Veterans General Hospital, Taipei, Taiwan; 7 Department of Medicine, National Yang-Ming University, Taipei, Taiwan; University of Southern California, United States of America

## Abstract

**Background:**

Pigs are an optimal animal for conducting biomedical research because of their anatomical and physiological resemblance to humans. In contrast to the abundant resources available in the study of mice, few fluorescent protein-harboring porcine models are available for preclinical studies. In this paper, we report the successful generation and characterization of a transgenic DsRed-Monomer porcine model.

**Methods:**

The transgene comprised a CMV enhancer/chicken-beta actin promoter and DsRed monomeric cDNA. Transgenic pigs were produced by using pronuclear microinjection. PCR and Southern blot analyses were applied for identification of the transgene. Histology, blood examinations and computed tomography were performed to study the health conditions. The pig amniotic fluid progenitor/stem cells were also isolated to examine the existence of red fluorescence and differentiation ability.

**Results:**

Transgenic pigs were successfully generated and transmitted to offspring at a germ-line transmission rate of 43.59% (17/39). Ubiquitous expression of red fluorescence was detected in the brain, eye, tongue, heart, lung, liver, pancreas, spleen, stomach, small intestine, large intestine, kidney, testis, and muscle; this was confirmed by histology and western blot analyses. In addition, we confirmed the differentiation potential of amniotic fluid progenitor stem cells isolated from the transgenic pig.

**Conclusions:**

This red fluorescent pig can serve as a host for other fluorescent-labeled cells in order to study cell-microenvironment interactions, and can provide optimal red-fluorescent-labeled cells and tissues for research in developmental biology, regenerative medicine, and xenotransplantation.

## Introduction

Rodents are the main laboratory animals used in establishing human disease models. However, research using rodents frequently yields findings that are not applicable to humans. Because of their anatomical and physiological resemblance to humans, pigs are an optimal model for preclinical research. Porcine models have been used in the pharmaceutical industry for the production of insulin, heparin, and coagulation factors. The completion of the swine genome [1] further facilitated genetic modifications for biomedical research and applications [2]. Genetically modified pigs can serve as donors to overcome the severe shortage of human organs for transplantation [3]. Several human clinical trials evaluating the therapeutic effects of using porcine xenotransplantation to treat type I diabetes mellitus (ClinicalTrials.gov NCT00940173), corneal disease (NCT01443559), and Parkinson's disease (NCT00226460) are ongoing. Pigs can also provide excellent sources of pluripotent stem cells for cell therapy.

Fluorescent proteins are critical for dissecting molecular and cellular processes in developmental biology, dynamically investigating gene expression and function in disease development, and tracking the fate of cells used in cell therapy and transplantation. In contrast to the ample resources available to researchers using fluorescent proteins of various wavelengths in rodents, fluorescent proteins available for researchers using porcine models remain under development. Our group [4] and Murakami et al. [5] have generated green fluorescent protein (GFP)-transgenic porcine models. Sommer et al. [6] produced transgenic pigs that expressed Stra8-enhanced yellow fluorescent protein. Matsunari et al. [7] successfully created Kusabira-Orange-expressing transgenic pigs. Recently, Lu et al. [8] created red-fluorescent transgenic Wuzhishan miniature pigs. However, reports of red-fluorescence-expressing large pigs with comprehensive phenotype characterization remain scarce. In this study, we investigated monomeric red fluorescent pigs as a potential model for biomedical research on human disease.

## Materials and Methods

The experiments were conducted in accordance with the Guide for the Care and Use of Agricultural Animals in Research and Teaching (Federation of Animal Science Societies 2010) and were approved by the Institutional Animal Care and Use Committee of National Taiwan University (NTU-96-EL-20).

### Construction of the DsRed transgene

**Figure 1:**
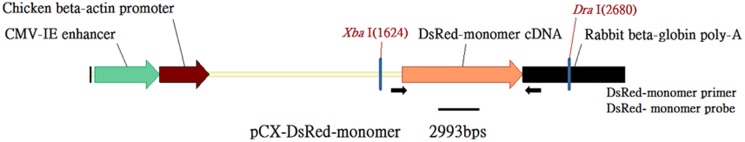
The DsRed-Monomer transgenic construct pCX-DsRed-Monomer. Arrows indicate the positions of the PCR DsRed primers. XbaI and DraI were restriction enzyme digestion sites. The thick black line indicates the position of the Southern blot probe.

The DsRed monomer coding sequence was cut from the pDsRed-Monomer-N1 Vector (Clontech Laboratories Inc., Mountain View, CA, USA) by using EcoRI digestion, treated with klenow, and used to replace the EGFP coding sequence between 2 EcoRI sites in pCX-EGFP [9]. The resulting plasmid was designated as the pCX-DsRed-Monomer, and contains a CMV enhancer, a chicken-beta actin promoter, a DsRed-Monomer cDNA fragment, and a rabbit beta-globin polyA site (Fig. 1).

### Production of transgenic pigs by using pronuclear microinjection

The transgenic pigs were generated by using procedures described previously [4]. Sexually mature crossbred gilts (Duroc ×
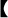
Landrace × Yorkshire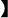) aged between 240 to 300 d and weighing greater than 120 kg were used as embryo donors or recipients. The animals' estruses were synchronized through daily oral administration of 20 mg of Regumate (Intervet, Millsboro, DE, USA) to the gilts for a period of 18 d. Donor animals were administered 2000 IU of pregnant mare's serum gonadotropin (PMSG) through injection (China Chemical and Pharmaceutical, Taipei, Taiwan) 24 h after the final dose of Regumate, and were injected with 1500 IU of hCG 80 h after being injected with the PMSG. Animals exhibiting standing estrus were artificially inseminated. Pronuclear embryos were collected from the oviduct 55–62 h after hCG injection.

The linearized transgene was purified from the Sal I and Hind III fragments of the pCX-DsRed-Monomer. The injected DNA was dissolved in a microinjection buffer (10 mM Tris, 0.1 mMEDTA, pH 7.4) at a concentration of 4 ng/uL. After microinjection, the pronuclear embryos were surgically transferred into recipient gilts on the same day.

### Identification of the transgene

Genomic DNA was extracted as described previously [4]. A PCR analysis of the genomic DNA was performed by primers specific for DsRed (forward, 5′-ATGGACAACACCGAGGACGTCATC-3′, and reverse, 5′- TCAGTGGTATTTGTGAGCCAGGGC-3′). Each reaction mixture contained 200 ng of genomic DNA extract, 0.1 µM of each primer, 1.25 units of Taq DNA polymerase (Invitrogen, Brazil) and 2.5 mM MgCl_2_, constituting a final volume of 20 uL. A PCR was conducted at 94°C for 3 min for 35 cycles (94°C, 30 s; 65°C, 30 s; and 72°C, 30 s), with a final extension at 72°C for 7 min. The amplified PCR product was separated on 1% agarose gel and exhibited the expected bands of 780 bp.

A Southern blot analysis was performed to confirm the transgene. The DNA were digested using XbaI and DraI (New England Biolabs, Beverly, MA, USA), separated in 1% agarose gel, and then transferred to a Hybond-N^+^ membrane (Amersham Biosciences) by using capillary diffusion. The blots were probed with the 232-bp PCR fragment of DsRed (forward, 5′- CGACATCCCCGACTACATGA-3′, and reverse, 5′- TCCTGGGGGTACAGCTTCTC-3′), which was [p^32^]dCTP-labeled by using the random primed labeling method. Hybridization was conducted under high stringency conditions (20% dextran sulfate, 2×SSC, 0.5% fat free milk powder, and 1% SDS) in the presence of 750 µg/mL of heat-denatured salmon sperm DNA. After a final wash with 2×SSC/0.1% SDS for 5 min at 65°C, the nylon membrane was sealed and exposed to an exposure cassette (Amersham Biosciences) with an intensifying screen at 4°C for 7 d.

### Histology and hematoxylin/eosin staining

Tissues were fixed in a 10% neutral formaldehyde solution overnight at 4°C, dehydrated through a series of graded ethanol baths, and then embedded in paraffin. The samples were sectioned at a thickness of 3 µm and stained with hematoxylin/eosin for analysis.

### Blood sample collection and measurements

Blood samples were collected in plastic vacutainer spray-coated K^2^EDTA tubes (Becton Dickinson, Franklin Lakes, NJ, USA) and lithium heparin vacuum tubes (Vacuette, Greiner Labortechnik, Kremsmunster, Austria) by using ear venipuncture. Routine blood measurements, including measurements of the red blood cell count, hemoglobin, hematocrit, white blood cell count, and platelet counts, were executed using the CELL-DYNE 1400 (Abbot Laboratories, Abbott Park, IL, USA). Blood biochemical parameters were analyzed using a Hitachi 7600 auto-analyzer (Hitachi Ltd, Tokyo, Japan), which also measured nutritional indices (total protein and albumin), renal function indices (blood urea nitrogen and creatinine), liver function indices (alanine aminotransferase, aspartate aminotransferase, and alkaline phosphatase), and metabolic indices (glucose, cholesterol, and triglyceride).

### Computed tomography

Whole-body computed tomography (CT) was performed to confirm the structural and functional integrity of the transgenic pigs. The pigs were anesthetized through an intramuscular injection of ketamine (12.5 mg/kg) and fentanyl (0.04 mg/kg). A 24-gauze catheter was subsequently inserted into the peripheral vein for contrast media infusion.

All images were acquired using a 256-slice multidetector CT scanner (Brilliance iCT; Philips Healthcare, Cleveland, OH, USA). Scanning was conducted at a tube voltage of 120 kV, a tube current of 120 mAs, a detector collimation of 128×0.625 mm, a 0.601 pitch, a gantry rotation of 270 ms, a field of view set to 20–25 cm, and a matrix size of 512×512. An iodinated contrast medium (2 ml/kg; Ultravist 370, Iopromide, Schering, Germany) followed by 5 mL of saline were infused through the venous catheter at 0.5 mL/sec. The contrast media and saline were infused, and after ensuring the opacification of the vasculature, scanning was immediately initiated from head to hoof, with simultaneous electrocardiographic recording. After scanning, the images were reconstructed with a slice thickness of 0.9 mm and a slice interval of 0.45 mm by using a standard algorithm (medium soft-tissue convolution kernel).

### Detection of red fluorescence

A portable light (excitation  = 520–540 nm, emission  = 580–650 nm) was used to detect red fluorescence in the pig. Paraffin-embedded sections of tissues were observed using a fluorescence microscope (Nikon Eclipse i80, Japan).

### Western blot analysis

Total proteins were extracted from snap-frozen tissues and separated on 10% SDS-PAGE gels. After being transferred to a PVDF membrane, the blots were probed with rabbit anti-DsRed polyclonal antibodies (1∶1500; BD Clontech, Palo Alto, CA, USA) and detected using HRP-conjugated goat anti-rabbit antibodies (1∶15,000; Bio-Rad) by using the ECL detection system (Amersham Bioscience).

### Isolation of pig amniotic fluid progenitor/stem cells

Pig amniotic fluid progenitor/stem cells (pAFPCs) were isolated from the Dsred pig at 70 d of pregnancy. The collected amniotic fluid was filtered through a 70-µm mesh (Miltenyi Biotec Inc., Auburn, CA, USA) and centrifuged at 12 000 rpm for 10 min. The pellets were resuspended and cultured in a 10-cm dish (TPP, Trasadingen, Switzerland) at a density of 2×10^5^ cells/cm^2^.

### Fluorescence-activated cell-sorting analysis

Ten to 12 passages of pAFPCs were treated using 0.25% trypsin EDTA for 5 min at 37°C, washed with a fluorescence-activated cell-sorting (FACS) buffer (PBS containing 2% fetal bovine serum), and then dissociated into single cells by gentle pipetting at a density of 1×10^6^ cells/mL. The cells were stained with fluorescein isothiocyanate (FITC)-conjugated mouse anti-pig antibodies CD4a, CD31, CD44, and CD90 (eBioscience Inc., San Diego, CA, USA) at 4°C for 30 min. The isotype was used as the negative control. The cells were analyzed using a FACS scan flow cytometer (Beckman Coulter Inc., Fullerton, CA, USA).

### Differentiation of pig amniotic fluid progentitor/stem cells

To evaluate the differentiation potency of the isolated pAFPCs, a series of in vitro differentiation experiments was conducted. To induce adipogenic differentiation, the pAFPCs were cultured in a StemPro Adipogenesis Supplement Medium (Gibco) for 3 wk; the differentiation medium was changed every 3 d. The pAFPCs were stained using 0.5% Oil-Red O (Sigma-Aldrich) to examine lipid droplet accumulation. For osteogenic differentiation, the pAFPCs were incubated in the StemPro Osteogenesis Supplement Medium (Gibco) for 1 wk, and the calcium deposition was confirmed by performing Alizarin red S (ARS; Sigma-Aldrich) staining. For chondrogenic differentiation, cell pellets were cultured in the StemPro Chondrogenesis Supplement Medium (Gibco) for 10 d. The production of proteoglycan was detected by performing toluidine blue (Sigma-Aldrich) staining.

### Statistical analysis

SPSS 15.0 (SPSS, Chicago, IL) was used to conduct statistical analysis. Groups were compared by using Student's *t* test for continuous variables. A *P* value less than 0.05 indicated statistical significance.

## Results

Sixty-five pronuclear embryos were injected with the DsRed gene, and 54 injected pronuclear embryos were transferred to 2 recipients. A total of 7 piglets were born, and 2 of the 7 harbored the DsRed gene. Our transgenic efficiency was comparable to that reported previously [10]. The germ-line transmission rate was approximately 43.59% (Table 1), which was concordant with Mendel's laws.

**Table 1:** Generation of red fluorescent protein (DsRed) transgenic pigs by pronuclear micro-injection.

**Donor pigs**	**3**
Pronuclear eggs	71
Microinjected pronuclear eggs	65
Embryos transferred to recipients	54
Recipient pigs	2
Pregnant recipients (%)	1 (50.0%)
Birth/embryos transferred (%)	7 (12.3%)
Transgenic survival pups (%)	2 (28.6%)
Germline transmission rate (%)	17/39 (43.59%)

### Expression of red fluorescence

**Figure 2:**
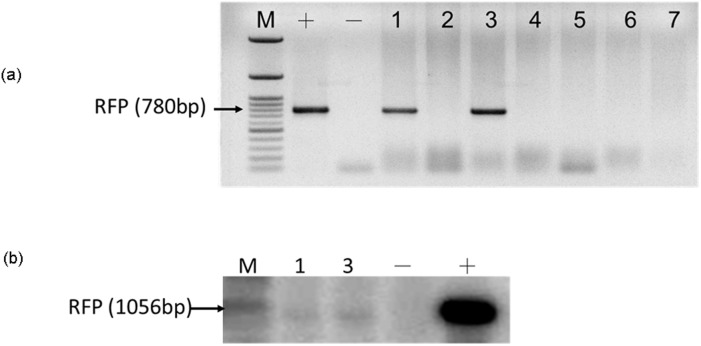
Transgenic founder pigs carrying the DsRed gene were detected by (a) PCR and (b) Southern blot analysis. The presence of transgenes in DsRed2 pigs were confirmed by using a DsRed primer, which produced a 780-bp PCR product. The genomic DNA of these 2 transgenic pigs (Nos. 1 and 3) were digested using XbaI and DraI. The digested DNA was subsequently hybridized using a 1.1-kb probe.

**Figure 3:**
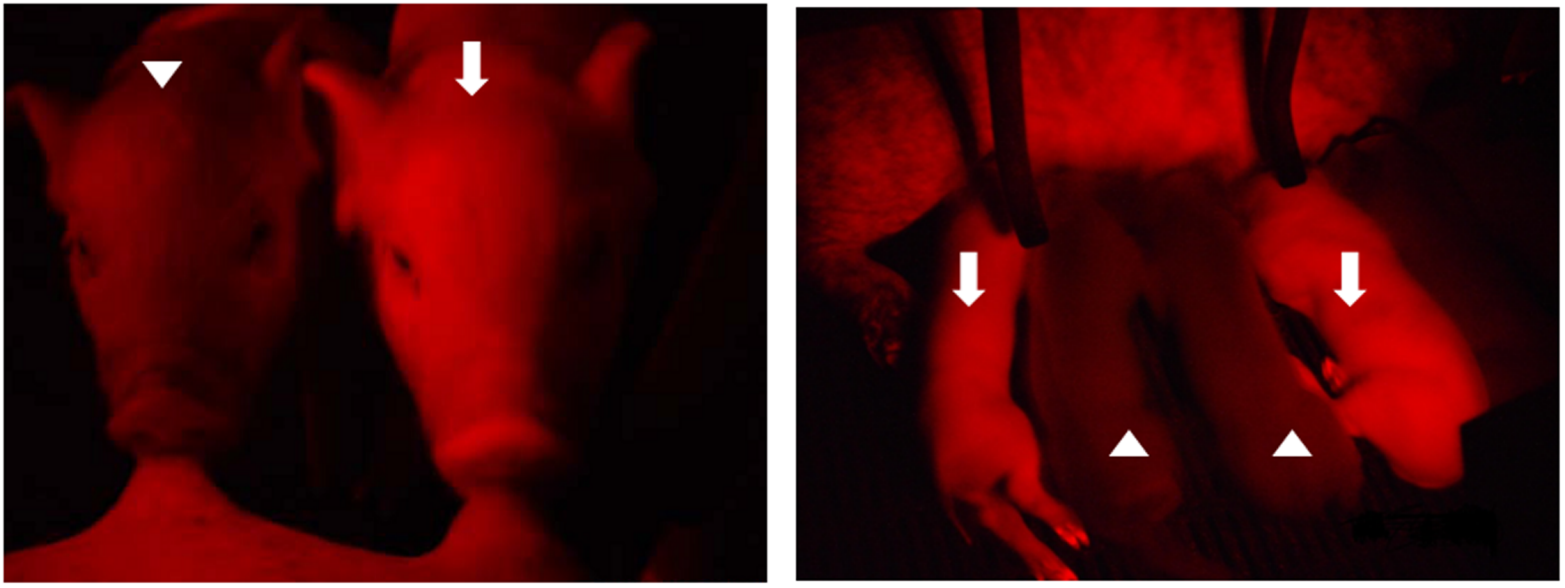
Transgenic piglets and (white arrow) and nontransgenic controls (white triangle) were differentiated according to the presence of external red fluorescence.

**Figure 4:**
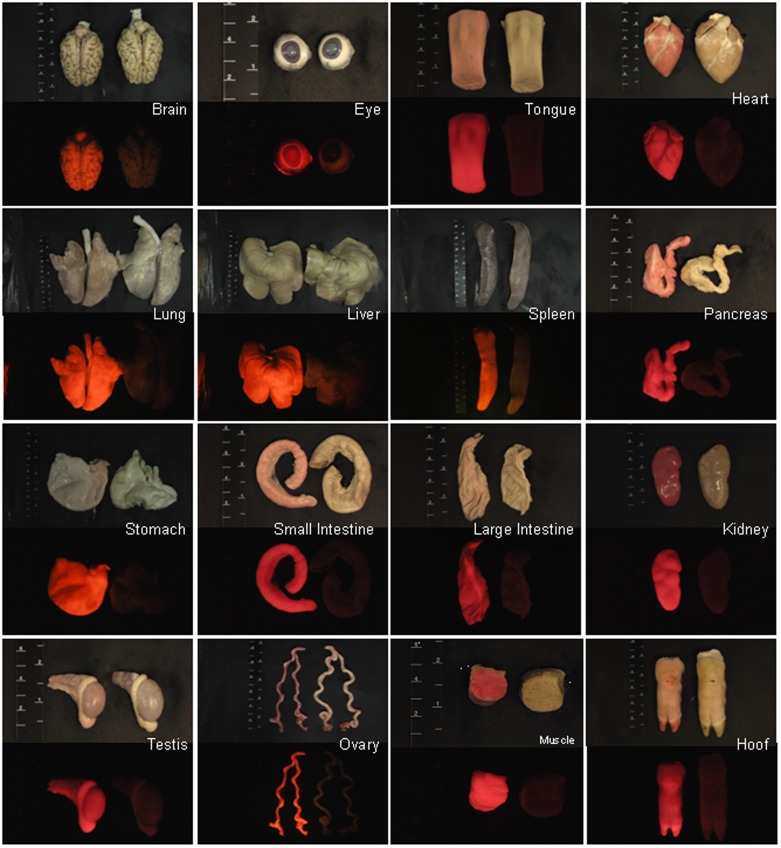
Bright-field images of tissues from the transgenic pig offspring (left) depict a distinct red fluorescence in comparison with the nontransgenic control (right).

**Figure 5:**
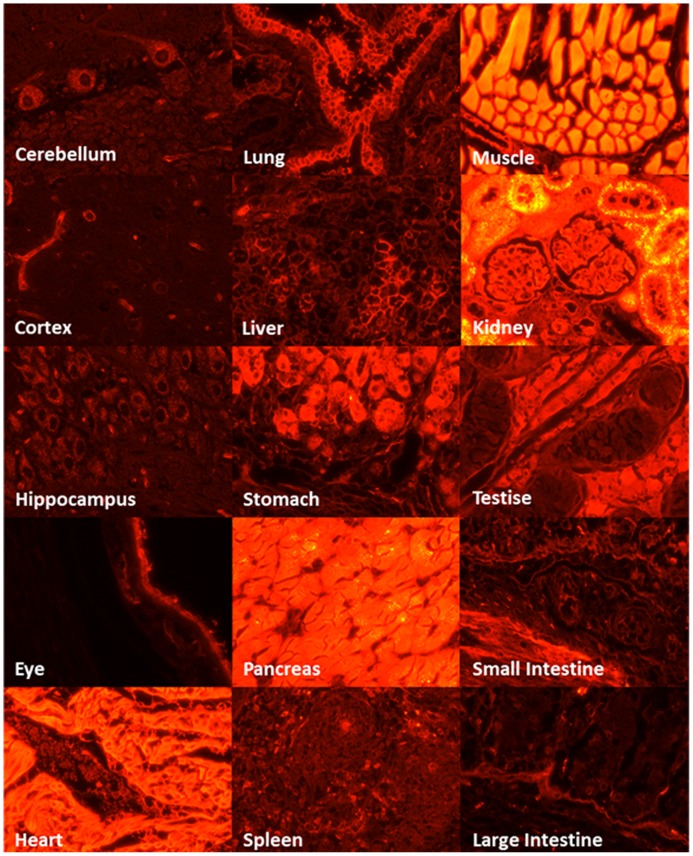
Fluorescence microscopy of the paraffin-embedded tissue sections of DsRed transgenic pigs revealed evident red fluorescence.

**Figure 6:**
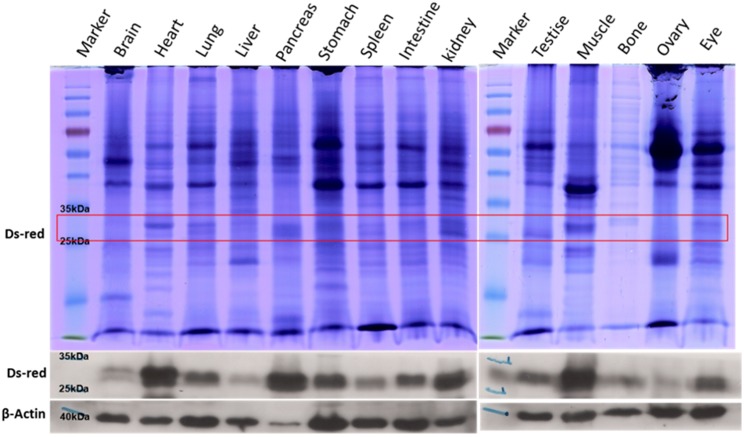
SDS-PAGE and western blot analysis of tissue samples from a DsRed transgenic pig.

Genome typing revealed that Piglets 1 and 3 carried the Ds-Red gene, and this was confirmed through a PCR (Fig. 2a) and Southern blotting (Fig. 2b). Fluorescent images depicted the ubiquitous exterior expression of red fluorescence in the transgenic pigs (Fig. 3). All of the harvested organs (Fig. 4) and tissues (Fig. 5), namely the brain, eye, tongue, heart, lung, liver, spleen, pancreas, stomach, small intestine, large intestine, kidney, testis, uterus, ovary, muscle, and hoofs, strongly exhibited red fluorescence. Hematoxylin and eosin staining revealed no obvious histological difference between the control and DsRed pigs. A western blot analysis indicated higher DsRed protein expression in the pancreas, heart, muscle, and kidney than the other organs (Fig. 6).

### Blood chemistry

Hematological analysis revealed no significant difference regarding white blood cell counts, hemoglobin levels, or platelet counts. Blood biochemical analysis revealed that the DsRed pigs did not differ from the control pigs in nutrition (as indicated by serum total protein and albumin levels), renal function (as indicated by blood urea nitrogen and creatinine levels), liver function (as indicated by alanine aminotransferase, aspartate aminotransferase, and alkaline phosphatase levels), or metabolic status (as indicated by glucose, cholesterol, and triglyceride levels) (Table 2).

**Table 2:** Blood routine and biochemistry between the transgenic offspring and the non-transgenic control pigs.

	Control N = 4	Transgenic N = 7	*P*
***Blood routine***			
Hemoglobin (g/dL)	10.68±0.74	11.83±1.08	0.146
Red blood cells (10^3^/mm^3^)	5.97±0.32	6.18±0.35	0.429
Mean corpuscular volume (fl)	55.58±2.91	59.86±3.42	0.113
Mean corpuscular hemoglobin (pg)	17.93±1.30	19.13±1.20	0.229
Mean corpuscular hemoglobin concentration (%)	32.20±0.75	31.97±0.43	0.609
White blood cells (/mm3)	9.99±2.05	10.26±2.13	0.869
Platelets (10^3^/mm^3^)	868.00±106.25	678.29±156.11	0.104
***Nutritional index***			
Total protein (g/dL)	5.70±0.21	5.73±0.18	0.832
Albumin (g/dL)	3.93±0.15	3.84±0.33	0.675
***Renal function index***			
Blood urea nitrogen (mg/dL)	8.15±2.92	9.11±1.72	0.548
Creatinine (mg/dL)	0.73±0.10	0.66±0.17	0.531
***Liver function index***			
Alanine aminotransferase (U/dL)	35.50±3.84	35.43±7.40	0.987
Aspartate aminotransferase (U/dL)	20.75±2.59	24.86±6.79	0.321
Alkaline phosphatase (U/dL)	281.50±36.53	248.14±67.29	0.428
***Metabolic index***			
Glucose (mg/dL)	124.00±15.31	126.43±18.53	0.845
Cholesterol (mg/dL)	103.75±32.41	131.71±42.03	0.326
Triglyceride (mg/dL)	61.25±35.50	57.86±50.61	0.917

### Computed tomography

There were no significant differences between the control and transgenic offspring regarding cardiac structure and function (Table 3).

**Table 3:** Cardiac structure and function between the transgenic offspring and the non-transgenic control pigs.

	Control N = 4	Transgenic N = 7	*P*
***Left ventricle***			
Left ventricle end systolic volume, mL	7.55±2.28	9.52±2.67	0.237
Left ventricle end diastolic volume, mL	23.85±4.87	26.6±4.87	0.374
Left ventricle stroke volume, mL	16.33±6.07	17.09±4.93	0.819
Left ventricle ejection fraction, %	66.25±12.63	63.25±9.49	0.652
Left ventricle cardiac output, mL	2573.6±1158.4	2284.2±690.1	0.594
Left ventricle mass, g	26.87±5.77	28.80±9.50	0.721
Heart rate, beats per minute	153.5±22.5	133.3±12.9	0.171
***Right ventricle***			
Right ventricle end systolic volume, mL	11.20±4.27	16.93±5.05	0.082
Right ventricle end diastolic volume, mL	26.37±5.96	32.08±5.23	0.119
Right ventricle stroke volume, mL	15.17±2.80	15.15±5.50	0.993
Right ventricle ejection fraction, %	57.75±9.63	46.50±13.87	0.180
Right ventricle cardiac output, mL	2342.4±648.1	1986.7±594.6	0.364

**Figure 7:**
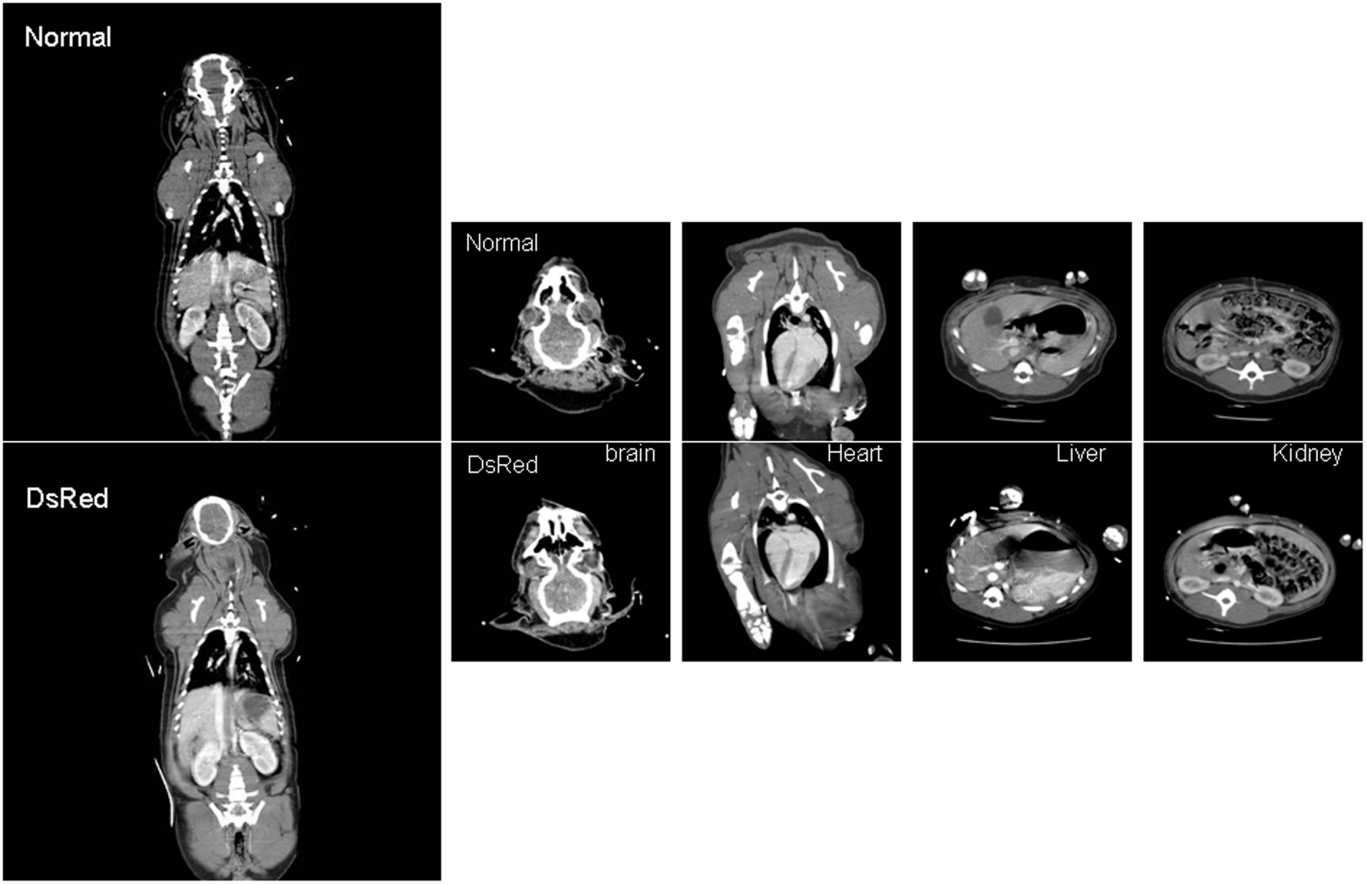
Whole-body CT of the normal and transgenic pigs. Neither structural differences nor abnormal lesions were observed in the DsRed pig.

The postcontrast enhanced CT scans showed no structural difference in the brain, no focal lesions in the liver and kidneys, well-distended gall bladders, no intrahepatic bile duct dilatations, no differences in parenchymal perfusion and density, and similar renal cortex thicknesses between the 2 groups (Fig. 7).

### Isolation, characterization, and differentiation of amniotic-fluid-derived progentitor/stem cells

**Figure 8:**
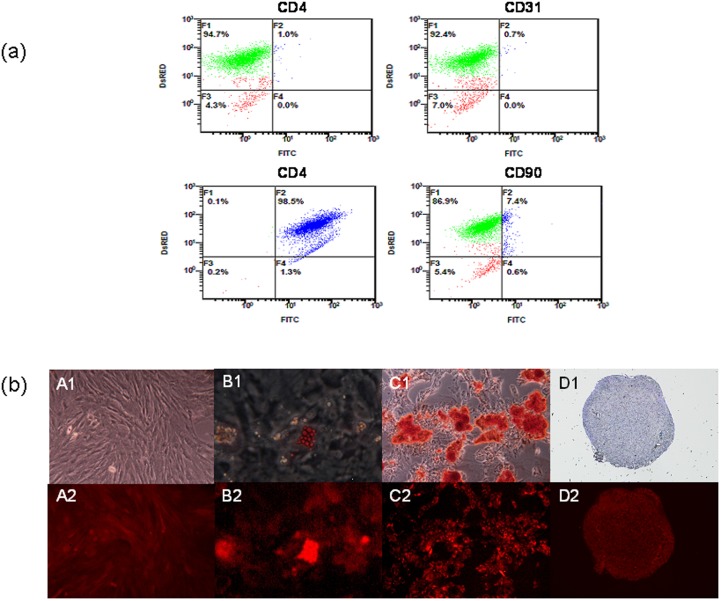
(a) Cytometric analyses of CD4a, CD31, CD44, and CD90 conjugated with FITC on DsRed pig amniotic fluid progenitor/stem cells (pAFPCs). pAFPCs were negative for the surface antigens CD4a and CD31, and positive for CD44 and CD90. (b) Mesoderm trilineage differentiation potential of DsRed pAFPCs. DsRed pAFPCs exhibited spindle-shaped morphology (A1) and expressed red fluorescence under fluorescent microscopy (A2). Lipid droplets were observed in the DsRed pAFPCs adipogenic differentiation culture by using Oil-Red O staining (B1). Calcium accumulation was observed in the osteogenic differentiation culture by using Alizarin red staining (C1). Collagen was detected in the chondrogenic differentiation culture by using toluidine blue staining (D1). Under fluorescent microscopy, red fluorescence was still evident after osteogenetic (B2), adipogenetic (C2), and chondrogenetic (D2) differentiation.

The pAFPCs isolated from our transgenic pigs expressed red fluorescence protein (Fig. 8A). The pAFPCs expressed the markers CD44 and CD90, but not CD4a or CD31. Of the pAFPCs, CD44 cells accounted for 98.5%, and CD90 cells accounted for 7.4% (Fig. 8). pAFPCs have the ability of adipogenic, osteogenic, and chondrogenic differentiation.

## Discussion

In this study, we successfully employed the pronuclear microinjection technique to produce pigs that ubiquitously expressed red fluorescent protein (RFP). Their health was comprehensively confirmed through blood biochemistry and whole-body CT. In research applications, the use of this animal enables red-fluorescent-protein-tagged cells to be isolated, and can be detected using live imaging in chimera studies. In addition, tagged tissues can facilitate research in developmental biology and xenotransplantation. In this study, we proved the differentiation potential of the animals' pAFPCs, which can serve as novel reagents for regenerative medicine with more effective cell-fate tracking.

The ability to monitor cell distribution, cell expression, and cell fate are fundamental to the development of cell therapy. Imaging of light emission from luminescent or fluorescent reporters enables real-time noninvasive in vivo analysis, and has become a mainstay of preclinical imaging [11], [12]. Traditional direct in vitro labeling techniques, such as DiI for labeling membranes, or Hoechst 33342 for labeling nuclei, are subject to dilution by cell proliferation. The dyes released by dead cells can label neighboring host cells and yield false-positive results [13]. Furthermore, these intercalating dyes can be toxic to cells, and might affect the differentiation potential of stem cells in vivo. Our red fluorescent pig can provide an ample source of cells for grafting without these potential confounding concerns, and can facilitate spatial, temporal, and quantitative analysis. Similar to our previous study on eGFP transgenic pigs [4], we observed various intensities of red fluorescence in different harvested organs and tissues. This phenomenon might be attributed to the differential assembly of transcription factors in each organ, which affected the spatial expression of our transgenic construct.

RFP was originally discovered in corals of the class Anthozoa [14]. This protein possesses 26%–30% of the conserved amino acid of aequorean GFP, and has a similar chromophoric structure. Unlike GFPs, the chromophore of DsRed contains an additional double bond that stretches its chromophore conjugation, resulting in its red color [15]. RFP offers the distinct advantage of low background autofluorescence [16]. However, the tight mutual binding of the 4 subunits in the original RFP causes any protein fused with the DsRed becomes a tetramer [17]. Such a tetramer interferes with protein-protein interactions, and even causes aggregation or precipitation [18]. This tetramer can be toxic to embryos [19] because it inhibits the expression of antiapoptotic proteins, such as the B-cell lymphoma extra-large protein, through translational regulation [20]. This is consequently the main hurdle in generating red fluorescence in transgenic animals. To solve the tetrameric stoichiometry issue, Campbell et al. [21] successfully generated monomeric RFP, which improved the reliability of protein fusion.

We comprehensively characterized our transgenic pig and employed hematology analysis, blood biochemistry profiling, and CT imaging to ensure the health of the pig. To date, we have observed no noticeable reduction of red fluorescence for over 5 generations. Moreover, in routine daily observations, no distinguishable behavioral differences between age-matched transgenic and control animals have been observed. In conjunction with various other fluorescent-tagged cells and organs, our red fluorescent pig forms the basis of a multicolored scheme for basic biomedical research on human diseases.

## Conclusions

We established and characterized a pig harboring the DsRed-Monomer. As a large domestic animal available for medical research, this pig is an addition to the armamentarium for elucidating many cellular processes in vivo, and can provide reagents for cell therapy and transplantation studies.
